# Reported Tetracaine Allergy in Cataract Surgery: A Phenotype-Based Framework for Preoperative Decision-Making

**DOI:** 10.3390/jcm15218241

**Published:** 2026-10-23

**Authors:** Margita Lucic, Sanja Petrovic Pajic, Tiana Petrovic, Dolika D. Vasovic, Jelena Mirkovic, Milica Jeremic, Marija Bozic

**Affiliations:** 1 Center for Anesthesiology and Resuscitation, University Clinical Center of Serbia, 11000 Belgrade, Serbia; 2 University Eye Hospital, University Clinical Center of Serbia, 11000 Belgrade, Serbia; 3 Faculty of Medicine, University of Belgrade, 11000 Belgrade, Serbia

**Keywords:** tetracaine, cataract surgery, local anesthetics, drug hypersensitivity, preoperative assessment, perioperative management, phenotypic framework

## Abstract

Reported tetracaine allergy, particularly in the absence of supporting medical documentation or a confirmed allergy diagnosis, represents a common challenge during the preoperative assessment of patients undergoing cataract surgery. In such cases, clinical decisions often rely primarily on patient history, which may be incomplete, inaccurate, or unreliable, resulting in considerable diagnostic uncertainty. A narrative review of the literature was conducted to examine the clinical relevance of reported tetracaine allergy in perioperative management of cataract surgery. Based on critical appraisal and synthesis of the available evidence, a phenotype-based framework comprising three clinical presentation phenotypes was developed, accompanied by a structured algorithm to support preoperative decision-making. The proposed framework classifies patients into three clinically relevant phenotypes: the irritative phenotype, the suspected hypersensitivity phenotype, and the clinical uncertainty phenotype. This classification provides a structured approach to interpreting reported tetracaine allergy and forms the basis of a practical algorithm for phenotype-guided preoperative management. The phenotype-based framework is intended to support, rather than replace, clinical judgment in the preoperative evaluation of patients with reported tetracaine allergy. By providing a structured approach to decision-making when diagnostic information is limited or uncertain, the framework may improve the consistency and transparency of perioperative management. Although conceptual, the proposed framework highlights the importance of obtaining reliable clinical information in patients reporting tetracaine allergy. Further methodological studies, including prospective validation, are required before the framework can be recommended for routine clinical practice.

## 1. Introduction

Cataract surgery is among the most frequently performed surgical procedures worldwide and is routinely undertaken under topical anesthesia in an ambulatory setting [1,2,3,4,5,6]. In this high-volume surgical setting, preoperative decision-making must be timely, evidence-based, and tailored to the individual patient’s risk profile. A particular challenge arises when patients report tetracaine allergy without supporting medical documentation or a confirmed allergy diagnosis [7,8,9,10,11,12,13,14]. In these circumstances, perioperative management frequently relies on patient-reported history, which may be incomplete, inaccurate, or remote, thereby creating substantial diagnostic uncertainty [15,16,17,18,19]. Unlike confirmed drug hypersensitivity, reported tetracaine allergy should be regarded as a clinical concern rather than an established diagnosis, requiring perioperative decision-making under conditions of considerable diagnostic uncertainty.

The evaluation of reported tetracaine allergy is further complicated by the indiscriminate use of the term *allergy* in routine clinical practice. The term is frequently applied to a broad range of adverse reactions that do not necessarily represent immune-mediated hypersensitivity [8,9,14,18]. Transient local irritation, burning sensations, conjunctival hyperemia, vasovagal episodes, anxiety-related symptoms, and other nonspecific reactions may all be misinterpreted as evidence of an allergic response [14,16,20]. Furthermore, limited evidence regarding the true incidence of tetracaine hypersensitivity, clinical manifestations, and potential cross-reactivity with other local anesthetics further contributes to diagnostic uncertainty [5,6,7,14,16].

In the absence of a structured approach to assessment, clinical management often varies considerably. Some patients undergo unnecessary postponement of surgery and referral for allergy evaluation, whereas others proceed directly to surgery without a systematic assessment of the reported reaction. This variability reflects the lack of a practical framework to support consistent and evidence-informed preoperative decision-making.

Although the clinical implications of reported tetracaine allergy are well recognized, the existing literature provides no structured model that links the characteristics and reliability of the reported reaction with an appropriate preoperative management strategy [3,18,20].

The aim of this review is to propose a phenotype-based framework for preoperative decision-making in patients reporting tetracaine allergy, developed through a critical appraisal of the available literature. The proposed framework integrates three clinically relevant phenotypes of presentation with a structured decision-making algorithm designed to standardize the preoperative assessment of these patients. It is intended to support, rather than replace, clinical judgment and allergy evaluation when indicated. By providing a systematic approach to managing diagnostic uncertainty, this framework may facilitate more consistent perioperative decision-making and serve as a foundation for future prospective clinical validation.

## 2. Materials and Methods

This study was conducted as a narrative review aimed at developing a clinically applicable phenotype-based classification and decision algorithm for the preoperative management of patients reporting tetracaine allergy. A narrative review methodology was considered appropriate because the objective of the study was not to quantitatively synthesize evidence or estimate treatment effects, but to develop a proposed conceptual framework by integrating heterogeneous evidence from the published literature through an iterative process of qualitative evidence synthesis and clinically informed conceptual refinement. The literature search was conducted from May to August 2026 using PubMed/MEDLINE, Scopus, and Google Scholar, without restrictions on publication year. Search terms included “tetracaine”, “topical ophthalmic anesthetics”, “local anesthetic allergy”, “ester local anesthetics”, “cross-reactivity”, “hypersensitivity”, “cataract surgery”, and “ophthalmic anesthesia”. These terms were combined using the Boolean operators AND and OR to identify publications addressing tetracaine hypersensitivity, local anesthetic cross-reactivity, and perioperative management in ophthalmic surgery. The final literature search was performed in August 2026. The review included original research articles, review papers, clinical guidelines, and relevant case reports addressing tetracaine allergy, hypersensitivity to local anesthetics, perioperative allergy assessment, cataract surgery and ophthalmic anesthesia. Articles published in English were considered eligible. Conference abstracts without sufficient methodological detail, duplicate publications, and articles not directly relevant to the objectives of this review were excluded. Given the limited number of publications that directly address this problem, the review was expanded to include the literature on hypersensitivity to local anesthetics, perioperative risk assessment, and ophthalmic anesthesia, including topical anesthetic use in cataract surgery [8,9,10,11,18,19,21,22,23,24,25]. Attention was given to identifying recurring patterns of clinical presentation relevant to preoperative decision-making. Publications were analyzed qualitatively with emphasis on the consistency of reported clinical manifestations, the level of diagnostic certainty, and their implications for perioperative management. Accordingly, the proposed framework should be interpreted as a conceptual synthesis of the available evidence intended to support clinical reasoning rather than as a validated evidence-based classification. These phenotypes were intended to reflect differences in clinical presentation and diagnostic certainty rather than distinct biological mechanisms of hypersensitivity. For the purposes of this conceptual framework, the term “phenotype” is used to describe clinically recognizable patterns of presentation and diagnostic certainty relevant to preoperative decision-making, rather than distinct biological or immunological phenotypes. Publications were compared according to the characteristics of the reported reaction, the level of diagnostic certainty, the availability of clinically relevant information, and the resulting implications for perioperative management. The three proposed phenotypic categories were developed by the authors through a clinically informed conceptual synthesis of the available evidence, integrating the characteristics of reported reactions with the reliability of the available clinical information. Throughout this process, particular attention was given to conceptual consistency and the potential clinical relevance of the proposed categories in routine ophthalmic anesthesia. Based on this classification, an accompanying decision algorithm was constructed by integrating the proposed phenotypes with evidence-informed perioperative management principles identified throughout the literature. The resulting framework is intended to provide a structured and transparent approach to individualized clinical decision-making in situations where diagnostic information is incomplete or uncertain. Because the objective of this review was conceptual model development rather than quantitative evidence synthesis, no formal assessment of methodological quality or risk of bias was performed. The proposed phenotype-based framework and accompanying decision algorithm represent a conceptual model intended to support preoperative decision-making and require prospective validation before being considered for routine clinical implementation.

## 3. Results: Development of the Conceptual Framework

### 3.1. Reported Tetracaine Allergy as a Source of Diagnostic Uncertainty

Reported tetracaine allergy represents a common source of diagnostic uncertainty in the preoperative assessment of patients undergoing ophthalmic surgery [2,5]. In most cases, neither medical documentation nor confirmed allergy diagnosis is available. Consequently, preoperative decision-making frequently relies almost exclusively on patient history, which is often incomplete, remote, or insufficiently detailed to permit reliable characterization of the reported reaction [3,6,16,17,18]. Unlike confirmed drug hypersensitivity, reported tetracaine allergy should be regarded as a clinical presentation of uncertain reliability rather than an established diagnosis. Reported reactions span a broad clinical spectrum, ranging from transient local irritation and expected pharmacological effects to symptoms potentially compatible with local or systemic hypersensitivity [4,8,10,26,27].

The clinical assessment is further complicated by anxiety-related symptoms, vasovagal reactions, and adverse effects associated with excipients or preservatives, all of which may mimic an allergic reaction [9,10]. Accordingly, the principal challenge lies not only in determining whether hypersensitivity is likely, but also in evaluating the reliability of the available clinical information on which perioperative decisions must be based. This inherent diagnostic uncertainty constitutes the rationale for the phenotype-based decision framework proposed in this study.

Taken together, the available evidence suggests that reported tetracaine allergy is more appropriately interpreted as a spectrum of clinical presentations than as a single diagnostic entity. This conceptual perspective provides the rationale for the proposed phenotype-based classification.

### 3.2. Phenotypic Classification of Reported Tetracaine Allergy

A critical appraisal of the available literature suggests that reported tetracaine allergy does not represent a single clinical entity but rather a heterogeneous spectrum of presentations associated with varying degrees of diagnostic certainty [8,9,10,11,12,14,15,16,17,18]. Accordingly, a simple binary distinction between *allergic* or *non-allergic* presentations is insufficient to guide rational preoperative management. Instead, we propose a phenotypic classification comprising three clinically relevant categories, as summarized in Table 1:

- Irritative phenotype;
- Suspected hypersensitivity phenotype;
- Clinical uncertainty phenotype.

Each phenotype reflects a distinct level of diagnostic certainty rather than a distinct biological mechanism and is intended to guide the corresponding perioperative management strategy.

The irritative phenotype represents patients whose clinical history is consistent with transient local symptoms commonly expected following topical anesthetic administration, without features suggestive of an immune-mediated reaction. Isolated, transient burning and conjunctival hyperemia occurring immediately after administration and resolving rapidly are more consistent with local irritation [3,8].

The suspected hypersensitivity phenotype applies to patients whose clinical history raises suspicion of a local or systemic allergic reaction, despite the absence of a confirmed diagnosis. Clinical features such as urticaria, angioedema, respiratory symptoms, or hypotension should raise suspicion of immediate hypersensitivity [1,3,9]. Delayed manifestations, including persistent itching, eyelid erythema, edema, or eczematous changes, may suggest delayed-type hypersensitivity [26]. The absence of systemic manifestations does not exclude localized allergic reactions. In these cases, the available information supports a more cautious preoperative strategy, including specialist allergy evaluation when feasible and consideration of an alternative local anesthetic or regional anesthesia when clinically appropriate.

The clinical uncertainty phenotype comprises patients for whom the available information is insufficient to reliably characterize the reported reaction. Incomplete history, absent medical documentation, or conflicting information may preclude classification into either of the preceding phenotypes. Rather than representing a distinct biological mechanism of reaction to tetracaine, this phenotype reflects the degree of diagnostic uncertainty facing the clinician during preoperative assessment rather than a distinct pathophysiological entity. Whenever additional clinical information becomes available, reassessment and reclassification should be undertaken before definitive preoperative planning. When diagnostic uncertainty cannot be resolved, perioperative management should be based on an individualized risk-benefit assessment.

Together, these three phenotypes constitute the foundation of a conceptual framework that links clinical assessment with preoperative decision-making. Their practical application is illustrated by the clinical management algorithm presented later in this manuscript.

**Figure 1:**
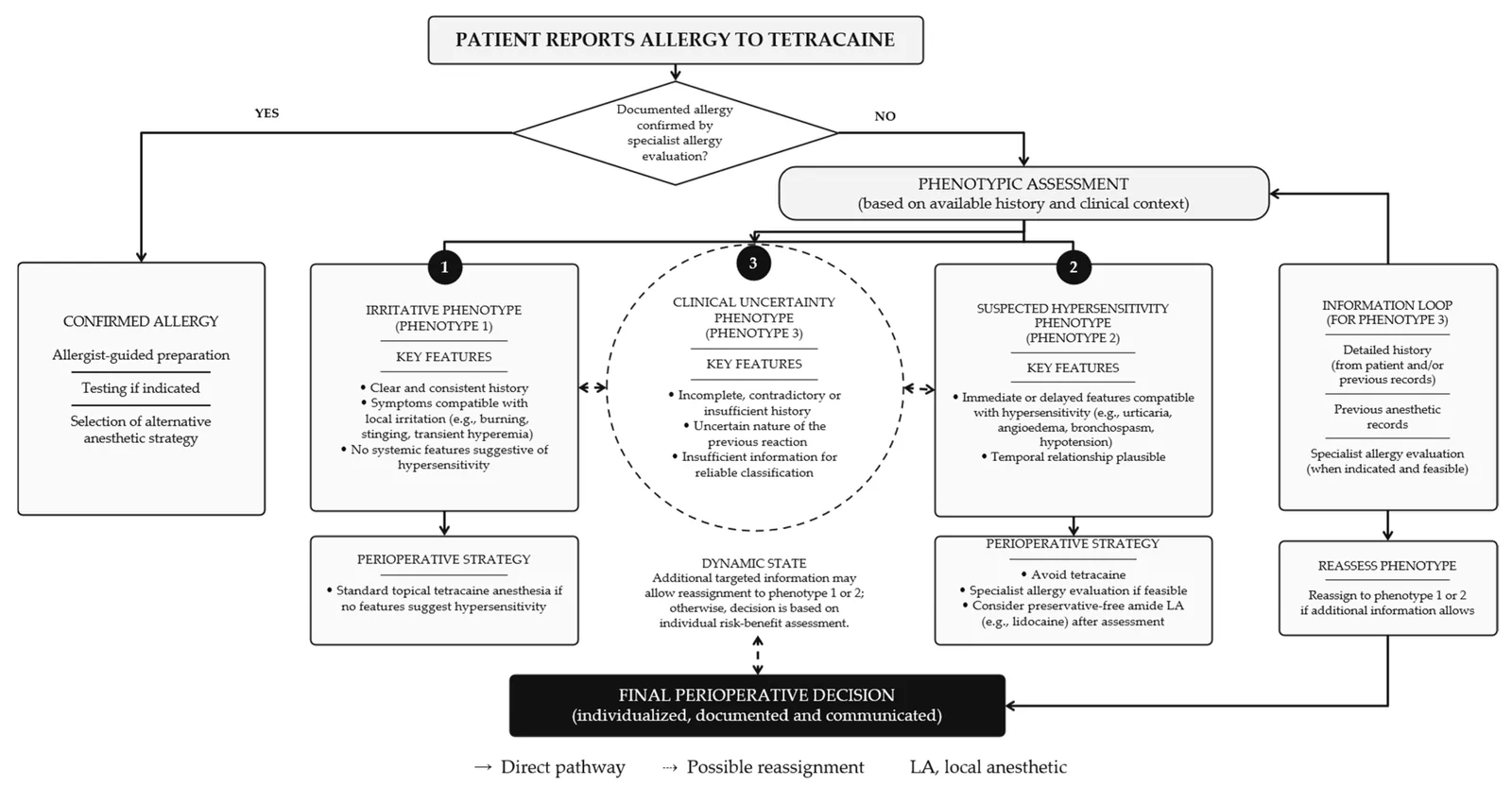
Proposed, non-validated phenotype-based conceptual framework for perioperative management of patients with reported tetracaine allergy.

The proposed classification serves as the conceptual basis for a phenotype-based approach to preoperative decision-making in patients reporting tetracaine allergy. Unlike the binary approach, where patients are seen strictly as “allergic” or “non-allergic”, the proposed framework introduces a degree of diagnostic certainty as an additional clinical criterion in the decision-making process. The phenotypic approach is not aimed at diagnosing an allergy but at structuring clinical thinking in situations where the available data are insufficient to reliably assess the nature of the reported reaction. This shifts the focus from confirming or ruling out an allergy to choosing the safest and most appropriate preoperative strategy based on the information available. In this way, the phenotypic classification serves as a bridge between patient history and preoperative decision-making. Each phenotype defines the level of diagnostic certainty, not the degree of allergic risk, allowing for a more consistent and rational approach to patients who report an allergy to tetracaine without proper medical documentation or a confirmed allergy diagnosis. The practical application of the proposed phenotype-based framework is shown in a clinical management algorithm (Figure 1), which operationalizes the phenotypic assessment into a structured preoperative decision-making process.

Patients with documented or confirmed tetracaine allergy follow an allergy-guided management pathway. In the absence of a confirmed diagnosis, reported reactions are classified into one of three phenotypes (irritative phenotype, suspected hypersensitivity phenotype, or clinical uncertainty phenotype) based on the available clinical history and the degree of diagnostic certainty. The clinical uncertainty phenotype is dynamic and may be reassessed if additional clinical information becomes available. Detailed clinical history-taking, review of available medical records, and individualized clinical judgement remain essential. The proposed algorithm is intended to structure this process, not to replace comprehensive clinical assessment or physician decision-making. When reclassification is not possible, perioperative management should be based on an individualized risk-benefit assessment.

## 4. Discussion

Local anesthetics have been used safely and effectively in clinical practice for more than a century and remain indispensable for surgical and diagnostic procedures [3,8]. They are broadly classified into ester (e.g., tetracaine, procaine, chloroprocaine, and benzocaine) and amide (e.g., lidocaine, mepivacaine, bupivacaine, and ropivacaine) compounds based on their chemical structure and metabolic pathways. Although true allergic reactions to local anesthetics are uncommon, hypersensitivity may occur to the anesthetic molecule itself or, more frequently, to preservatives or other excipients contained in the formulation. In this review, hypersensitivity refers to clinical reactions suggestive of an immunologically mediated response, including immediate and delayed reactions, irrespective of whether the underlying mechanism has been confirmed by formal allergy testing. This term is distinguished from non-allergic adverse reactions, such as local irritation, pharmacological effects, and anxiety-related or vasovagal symptoms. Clinical manifestations range from localized cutaneous reactions to, rarely, systemic anaphylaxis.

Tetracaine (amethocaine), an ester local anesthetic, is among the most used topical anesthetics in ophthalmic surgery. Although both immediate hypersensitivity reactions and delayed allergic contact dermatitis have been described, confirmed tetracaine allergy remains uncommon. Immediate and delayed reactions require different diagnostic approaches, which should be selected according to the clinical presentation and timing of symptoms. Nevertheless, patients reporting tetracaine allergy in the absence of supporting medical documentation or formal allergy testing represent a clinically important challenge in routine ophthalmic practice. Because cataract surgery is typically performed as an elective ambulatory procedure, perioperative decisions frequently must be made immediately before surgery, often based on incomplete or unreliable clinical history [2,3,6]. Before a final perioperative decision is made, the available clinical information should be critically reassessed, with particular attention to the characteristics of the reported reaction, its temporal relationship to tetracaine exposure, and the reliability of the patient’s history. When clinically indicated and feasible, additional medical documentation or specialist allergy evaluation should be obtained. If diagnostic uncertainty persists, the decision should be individualized, taking into account the severity of the suspected reaction, the available anesthetic alternatives, and the risks associated with proceeding without diagnostic clarification.

Although the available literature describes the epidemiology, clinical manifestations, and potential cross-reactivity of local anesthetics, it provides little guidance for preoperative management when the nature of a reported allergic reaction cannot be reliably established [8,16,18,28,29,30,31,32,33,34]. The phenotype-based framework proposed in this study was developed to address this gap by providing a structured approach to clinical decision-making under conditions of diagnostic uncertainty.

Tetracaine belongs to the ester group of local anesthetics, which also includes procaine, chloroprocaine, and benzocaine. When tetracaine hypersensitivity is suspected, potential cross-reactivity with other ester local anesthetics should be considered, particularly in patients with a history suggestive of allergic contact sensitization [14,16,26]. Amide local anesthetics, such as lidocaine, may represent a reasonable alternative because cross-reactivity between ester and amide agents is generally considered uncommon [3,14,16]. However, chemical classification alone does not establish the safety of an alternative agent. Reactions attributed to local anesthetics may also involve formulation components, including preservatives such as methylparaben [3,8]. Therefore, anesthetic selection should be guided by the clinical history, the specific formulation, and specialist allergy evaluation when indicated [3,8]. In a published case report, preservative-free lidocaine was successfully used for cataract surgery following negative allergy testing with the preservative-free formulation in a patient with reported local anesthetic allergy [27]. In patients with a history suggestive of severe immediate hypersensitivity or anaphylaxis, formal allergy assessment before elective surgery should be considered whenever feasible [3,32,33].

A central premise of the proposed framework is that, in the absence of a confirmed diagnosis, the quality and reliability of the available clinical information may be more relevant to perioperative decision-making than the presumed immunological mechanism of the reported reaction. Accordingly, the proposed classification deliberately integrates two distinct dimensions: the pattern of the reported reaction and the reliability of the available clinical history. While the irritative and suspected hypersensitivity phenotypes are primarily defined by the reported clinical presentation, the clinical uncertainty phenotype reflects insufficient or unreliable information rather than a distinct reaction type. This approach better reflects real-world perioperative practice, where decisions frequently depend on incomplete clinical information rather than definitive diagnostic testing.

A particular strength of the proposed model is the introduction of the clinical uncertainty phenotype. Unlike the irritative and suspected hypersensitivity phenotypes, this category does not describe a distinct biological response to tetracaine. Instead, it explicitly recognizes situations in which the available information is insufficient to reliably characterize a previous reaction. By identifying diagnostic uncertainty as a distinct clinical category, the framework provides a transparent rationale for preoperative decision-making and may help reduce unwarranted variability in clinical practice. Importantly, this phenotype should be regarded as a dynamic clinical category rather than a definitive classification, allowing reassessment whenever additional information becomes available.

The accompanying clinical algorithm represents the practical application of this conceptual framework. Rather than attempting to diagnose or exclude allergic hypersensitivity, it guides clinicians through a structured assessment of the available evidence and facilitates selection of an appropriate perioperative management strategy according to the level of diagnostic certainty. Its purpose is not to replace clinical judgment or specialist allergy evaluation when indicated, but to promote a more consistent and reproducible approach to patients with reported tetracaine allergy. In this way, the algorithm translates a conceptual framework into a practical tool that may improve consistency of perioperative management across different clinical settings.

Several limitations should be acknowledged. The proposed phenotype-based framework and decision algorithm are conceptual and were developed through a narrative review and synthesis of the available literature rather than prospective clinical investigation. Furthermore, the proposed phenotypes have not yet undergone formal interobserver validation, and their reproducibility in routine clinical practice remains to be established. Prospective multicenter studies are needed to evaluate the reliability of the phenotypic classification, validate the proposed algorithm, and determine whether its implementation improves the consistency and quality of preoperative decision-making while reducing unnecessary variability in clinical practice.

## 5. Conclusions

This study addresses an important challenge in the perioperative management of patients reporting tetracaine allergy in the absence of supporting medical documentation or a confirmed diagnosis. In these situations, clinicians must often determine the safest approach to cataract surgery based on incomplete and potentially unreliable historical information.

The proposed phenotype-based framework classifies reported tetracaine reactions into three clinically relevant phenotypes and provides a structured algorithm to support preoperative assessment. Rather than diagnosing allergy, the framework is intended to support clinical judgement by integrating the quality and reliability of the available clinical information into perioperative planning.

Although conceptual, the proposed framework highlights the importance of obtaining reliable clinical information in patients reporting tetracaine allergy. Further methodological studies, including prospective validation, are required before the framework can be recommended for routine clinical practice. If prospectively validated, the proposed framework may contribute to more consistent, transparent, and individualized preoperative decision-making in patients reporting tetracaine allergy.

## Figures and Tables

**Table 1:** Phenotype-based classification of reported tetracaine allergy.

Characteristic	Phenotype 1 *Irritative*	Phenotype 2 *Suspected Hypersensitivity*	Phenotype 3 *Clinical Uncertainty*
**Clinical history**	Clear and consistent	Clear and consistent	Incomplete, contradictory, or insufficient for a reliable assessment
**Pattern of clinical presentation**	Local irritation, transient burning sensation, transient conjunctival hyperemia, or other expected local reaction	Immediate (urticaria, angioedema, respiratory symptoms, hypotension) or delayed eczematous reactions, temporally compatible with tetracaine exposure	The nature of the previous reaction cannot be reliably determined
**Clinical dilemma**	Distinguishing an irritant reaction from an allergic reaction	Estimating the likelihood of hypersensitivity	Decision-making under diagnostic uncertainty
**Recommended** **preoperative strategy**	Proceed with standard topical tetracaine anesthesia after excluding features suggestive of hypersensitivity	Specialist allergy evaluation, when feasible. Consider preservative-free amide local anesthetic (e.g., lidocaine) after appropriate assessment	Obtain additional clinical information and reassess whenever feasible. Consider allergy evaluation if uncertainty persists. Individualize the final decision

Note. The clinical uncertainty phenotype represents a temporary category pending acquisition of additional clinical information. Whenever sufficient information becomes available, patients should be reassessed and reclassified as Phenotype 1 or Phenotype 2, as appropriate. When reclassification is not possible, the final preoperative decision should be based on an individualized risk-benefit assessment.

## Data Availability

No new data were created or analyzed in this study. Data sharing is not applicable to this article.
